# Antimicrobial Resistance of Major Bacterial Pathogens from Dairy Cows with High Somatic Cell Count and Clinical Mastitis

**DOI:** 10.3390/ani11010131

**Published:** 2021-01-08

**Authors:** Reta D. Abdi, Barbara E. Gillespie, Susan Ivey, Gina M. Pighetti, Raul A. Almeida, Oudessa Kerro Dego

**Affiliations:** 1 Department of Animal Science, Hebert College of Agriculture, The University of Tennessee, Knoxville, TN 37996, USA; Reta.Abdi@liu.edu (R.D.A.); bgillesp@utk.edu (B.E.G.); ivey@utk.edu (S.I.); pighetti@utk.edu (G.M.P.); ralmaida@utk.edu (R.A.A.); 2 Department of Biomedical Sciences, College of Veterinary Medicine, Long Island University Post, Roth Hall, Brookville, NY 11548, USA

**Keywords:** antimicrobial resistance, dairy cow, mastitis pathogen, intramammary infection, environmental pathogen, contagious pathogen

## Abstract

**Simple Summary:**

Mastitis is the most prevalent disease of dairy cattle that causes significant economic losses. Different agents cause mastitis which leads to increased somatic cell count (SCC) and low milk quality. Treating mastitis with antimicrobials is essential to reduce SCC and improve milk quality. Excessive use or misuse of antimicrobials in dairy farms leads to the development of antimicrobial resistant bacteria. The objectives of this study were (1) to isolate and identify the causative agent of mastitis and (2) determine antimicrobial resistance profiles of bacterial isolates. A total of 174 quarter milk samples from 151 cows with high SCC and clinical mastitis from 34 dairy farms in Tennessee, Kentucky, and Mississippi were collected. Bacterial causative agents were determined by bacteriological and biochemical tests. Antimicrobial resistance of bacterial isolates against 10 commonly used antimicrobials was tested. A total of 193 bacteria consisting of six bacterial species, which include *Staphylococcus aureus*, *Streptococcus uberis*, *Streptococcus dysgalactiae*, *Escherichia coli*, *Klebsiella oxytoca* and *Klebsiella pneumoniae* were isolated. *Staphylococcus aureus* was the predominant isolate. The proportion of resistant isolates was relatively higher in Gram-negatives than Gram-positives. Continuous antimicrobial resistance testing and identification of reservoirs of resistance traits in dairy farms are essential to implement proper mitigation measures.

**Abstract:**

Mastitis is the most prevalent and economically important disease caused by different etiological agents, which leads to increased somatic cell count (SCC) and low milk quality. Treating mastitis cases with antimicrobials is essential to reduce SCC and improve milk quality. Non-prudent use of antimicrobials in dairy farms increased the development of antimicrobial resistant bacteria. This study’s objectives were (1) to isolate and identify etiological agents of mastitis and (2) to determine antimicrobial resistance profiles of bacterial isolates. A total of 174 quarter milk samples from 151 cows with high SCC and clinical mastitis from 34 dairy farms in Tennessee, Kentucky, and Mississippi were collected. Bacterial causative agents were determined by bacteriological and biochemical tests. The antimicrobial resistance of bacterial isolates against 10 commonly used antimicrobials was tested. A total of 193 bacteria consisting of six bacterial species, which include *Staphylococcus aureus*, *Streptococcus uberis*, *Streptococcus dysgalactiae*, *Escherichia coli*, *Klebsiella oxytoca* and *Klebsiella pneumoniae* were isolated. *Staphylococcus aureus* was the predominant isolate followed by *Strep.* spp., *E. coli*, and *Klebsiella* spp. Results of this study showed that Gram-negatives (*E. coli* and *Klebsiella* spp.) were more resistant than Gram-positives (*Staph. aureus* and *Streptococcus* spp.). Continuous antimicrobial resistance testing and identification of reservoirs of resistance traits in dairy farms are essential to implement proper mitigation measures.

## 1. Introduction

Bovine mastitis causes significant economic losses to dairy farms and usually results in a loss of sustainability of dairy farming [1,2]. Dairy cows are particularly susceptible to intramammary infection (IMI) during early non-lactating (dry period) and transition periods [3,4]. The incidence of IMI is high during the early dry period because of the cessation of hygienic milking practices such as pre-milking teat washing and drying [5] and pre-and post-milking teat dipping in antiseptic solutions [6,7], which are known to reduce teat end colonization by bacteria and thus, infection. Udder infected during the early dry period usually persists and manifests clinical mastitis during the transition period [8] because of increased production of parturition inducing immunosuppressive hormones [9], negative energy balance [4], and physical stress during calving [10].

Over 135 various microorganisms have been identified from bovine mastitis, of which the most common bovine mastitis pathogens are commonly classified as either contagious or environmental mastitis pathogens [11]. Major contagious mastitis pathogens include *Staphylococcus aureus*, *Streptococcus agalactiae*, and *Mycoplasma bovis* [12,13]. Environmental mastitis pathogens include a wide range of organisms, including coliforms (*Escherichia coli*, *Klebsiella*, *Enterobacter*, and *Citrobacter*), environmental streptococci (*Streptococcus uberis* and *Streptococcus dysgalactiae*), *Trueperella pyogenes*, non-aureus staphylococci (NAS) and others such as *Pseudomonas*, *Proteus*, *Serratia*, *Aerococcus*, *Listeria*, yeast, and *Prototheca* [12,14] species.

The IMI may progress to inflammation and mastitis, leading to increased somatic cell count (SCC) in milk to fight off infection. Thus, SCC is an indicator of milk quality [15,16,17,18,19]. The milk quality of dairy farms can be estimated using bulk tank milk somatic cell count (BTSCC) for setting a premium price for quality milk producers. Grade A milk category in the USA has the maximum SCC of 750,000 cells/mL of bulk tank milk, but most processing and marketing organizations require lower than 400,000 cells/mL. In fact, California, Oregon, Idaho, and Washington have already lowered the maximum SCC of Grade A milk to 600,000, 500,000, 400,000, and 400,000 cells/mL of bulk tank milk, respectively [20]. In a healthy udder, SCC ranges from 50,000 to 100,000 cells/mL in which SCC < 200,000 cells/mL is the cut-off value for composite milk from all quarters of a healthy cow whereas SSC > 200,000 cells/mL is the cut-off value for a cow with IMI, but cows are only treated with antimicrobials when their SCC exceeds 250,000 cells/mL of udder milk [21]. To reduce the high BTSCC of a farm, cases of mastitis should be treated and cured or, if not curable by treatment, should be culled.

To prevent IMI during the dry period, in the United States and many other countries at the end of lactation (at drying off), dairy cows are given an intramammary infusion (IMIF) of long-acting antimicrobials (blanket dry cow therapy—BDCT) [22,23] or selective dry cow therapy (SDCT) [24,25]. Under BDCT, all udder quarters of all cows are infused with an antimicrobial, whereas under SDCT, IMIF of an antimicrobial is given only for quarters with high SCC. In addition, antimicrobials are also used for the treatment of mastitis [22,23] and other diseases of dairy cattle such as metritis, retained placenta, lameness [26,27,28], pneumonia [29,30,31], and neonatal calf diarrhea [32]. Some farms also feed raw waste milk or pasteurized waste milk from antimicrobial-treated cows to calves, which increases pressure on gut microbes to become antimicrobial-resistant [33,34,35]. Some of the antimicrobials used in dairy farms include beta-lactams (penicillins, ampicillin, oxacillin, penicillin-novobiocin), extended-spectrum beta-lactams (third generation cephalosporins, e.g., ceftiofur), aminoglycosides (streptomycin), macrolides (erythromycin), lincosamide (pirlimycin), tetracycline, sulfonamides, and fluoroquinolones [36,37,38]. Antimicrobials are administered to dairy cows mainly through intramuscular and intramammary routes [39]. Exposure of large numbers of animals in dairy farms to antimicrobials, exerts intense selective pressure on microbes in the body of animals and farm environments to become resistant to antimicrobials. Monitoring antimicrobial resistance (AMR) patterns of bacterial isolates from mastitis cases and farm environments are essential not only for treatment decisions but also to determine potential reservoirs of resistome in dairy farms.

Milk processing and marketing organizations prefer milk with low SCC (<200,000 cells/mL), and farms with low SCC sell their milk at a premium price, but farmers seem to struggle to lower the SCC threshold of their farms. The increased non-prudent antimicrobials usage practices in dairy farms seem part of the farmers’ efforts to eliminate bacteria from the udder to reduce the SCC of their farms. Cows with high SCC (>200,000 cell/mL) are most likely to have a high bacterial load, suffer from subclinical mastitis, and serve as a reservoir of pathogenic bacteria for healthy cows. Farmers usually never notice cows with high SCC, and they remain in lactation without being treated. Farmers usually administer antimicrobials to cows with clinical mastitis without prior antimicrobial sensitivity testing, but the cure rate is low, especially for chronic cases. Those cows with chronic infection most likely serve as a source of antimicrobial-resistant bacteria. Therefore, the objectives of this study were (1) to isolate and identify bacterial etiological agents from dairy cows with SCC of above 200,000 cells/mL and from cows with clinical mastitis and (2) to determine antimicrobial resistance profiles of bacterial isolates.

## 2. Materials and Methods

### 2.1. Study Farms and Animals’ Selection

To enroll farms in this study, first, the total number of dairy farms in each state were identified. Second, based on monthly BTSCC from Dairy Herd Improvement (DHI), farms that have BTSCC > 200,000 cells/mL of milk were enrolled in this study through volunteer participation of producers (*n* = 34). After participating farms were identified, the SCC of cows in each participating farm was determined to identify cows with high SCC (>200,000 cells/mL). Individual quarter milk samples were collected from a maximum of twenty cows with high SCC (>200,000 cells/mL) randomly and from cows that developed clinical mastitis during study time. The maximum total number of cows per farm was set to 20 cows because of expenses associated with this study. In this study, clinical mastitis was defined as an infection of one or more udder quarters of a cow that manifested visible inflammatory changes such as redness, swelling, pain, increased heat and/or visible inflammatory changes in milk (watery, bloody, blood-tinged, serum-like, etc.) or consistency (clots or flakes or stringy or viscous). Subclinical mastitis was defined as an infection of one or more udder quarters of a cow without manifesting visible inflammatory changes in the mammary gland tissue or milk but with high SCC (>200,000 cells/mL of milk). The SCC was determined at the Dairy Herd Improvement Association (DHIA) Laboratory (Knoxville, TN, USA) using the Soma Count 300 (Bentley Instruments Inc., Chaska, MN, USA).

### 2.2. Milk Samples Collection

In total, 174 quarter milk samples were collected from 151 cows from 34 dairy farms in Kentucky, Mississippi, and Tennessee. Approximately 10 mL of quarter milk samples were collected in sterile plastic tubes aseptically. Each cow’s teat pre-dipped into antiseptic solution, dried with an individual paper towel, and the teat opening was scrubbed with 70% alcohol. Individual quarter milk sample was collected after the first 2–3 squirts were stripped out to remove contaminant bacteria from the teat canal. For each sample, additional farm data were recorded, which include a herd identification number, cow identification number, a quarter of a cow, and status of a quarter (clinical or subclinical). Samples were collected aseptically by researchers and kept on ice and transported to the Tennessee Quality Milk Laboratory at the University of Tennessee, Knoxville, TN, to isolate and identify bacteria within 1–2 h of collection.

#### 2.2.1. Bacterial Isolation and Identification

Bacteriological culturing of milk, isolation, and identification of causative bacterial pathogens was conducted following the National Mastitis Council guideline [40] with some modification as described elsewhere [15,19,41,42]. Briefly, 100 µL of milk sample was inoculated on trypticase soy agar supplemented with 5% sheep blood (blood agar plates) (Becton, Dickinson Co, Sparks, MD, USA) and incubated at 37 °C with 5% CO_2_:95% air incubator for 24–48 h. The plates were examined for bacterial growth at 24 and 48 h of incubation. Milk samples with the growth of three or more colony types were considered contaminated during collection and discarded and re-collected. Milk samples with two different colonies were considered a mixed infection. The type of hemolysis (alpha, beta, double, and gamma) was determined on blood agar plates. Each visible colony was Gram-stained and differentiated into Gram-positive or -negative organism with morphological characterization. A catalase test was conducted on Gram-positive cocci to differentiate staphylococci from streptococci. Staphylococci are catalase-positive, whereas streptococci are catalase-negative. Catalase-positive staphylococci were further differentiated into coagulase-positive and -negative by tube coagulase test using rabbit plasma and API Staph strip (BioMetrieux Inc., Durham, NC, USA). Catalase negative cocci were further evaluated by API Strep (BioMetrieux Inc). The oxidase test was utilized to differentiate the *Enterobacteriaceae* from Gram-negative non-*Enterobacteriaceae* organisms. *Enterobacteriaceae* are oxidase negative. Oxidase negative members of *Enterobacteriaceae* were further inoculated to McConkey agar and tested by API strip for Gram-negative bacilli (BioMetrieux Inc.).

#### 2.2.2. Antimicrobial Sensitivity Test

Antimicrobial resistance patterns of each isolate were tested as described elsewhere [43] by minimum inhibitory concentration (MIC) using the broth microdilution method on commercially prepared 96-well microtiter plates from Sensititre system (Thermo Fisher Scientific, Cleveland, OH, USA) according to clinical laboratory standard institute guidelines (CLSI 2018) [44]. The Sensititre system had a panel of 10 commonly used antimicrobials. The 10 antimicrobials tested in the panel with their concentration (μg/mL) include ampicillin (0.12–8), penicillin (0.12–8), erythromycin (0.25–4), oxacillin +2% NaCl (2–4), pirlimycin (0.5–4), penicillin-novobiocin (P/N) combination (8/16–½), tetracycline (1–8), cephalothin (2–16), ceftiofur (0.5–4), and sulfadimethoxine (32–256).

### 2.3. Data Analysis

Multivariable logistic regression analysis was conducted to predict the occurrence of clinical and subclinical mastitis among three US states and four udder quarters with six bacterial etiological agents. Cases of subclinical and clinical mastitis were assumed as an output or response (dependent) variable. Accordingly, subclinical and clinical cases were represented with 0 and 1, respectively, for multivariable logistic regression analysis. Three US states, udder quarters, bacterial etiological agents, and farms, were considered as independent predictor (input) variables for the occurrence of subclinical or clinical mastitis. The states, udder quarters, and bacterial etiological agents were included as a predictor variable with a fixed effect in the General Linear Model to conduct analysis by Generalized Estimation Equation (GLM-GEE) binary logistic analysis model. The type of bovine mastitis (clinical or subclinical) was included in the model as a response variable.

The MIC values of the isolates were summarized by descriptive statistics using the median and its 95% error bars. According to CLSI 2018, each bacterial isolate can be classified into one of the three groups (susceptible, intermediate, and resistant) based on its response to each antimicrobial agent as measured by MIC. However, if the number of isolates in the resistant group is very low, the CLSI 2018 guideline recommends the merging of both intermediate and resistant isolates into one (non-susceptible) group, resulting in only susceptible and non-susceptible groups. Thus, we used the second CLSI 2018 option due to the low number of isolates in the resistant group for some of the bacterial isolates. Subsequently, the MIC values of each antimicrobial against each bacterial isolate were classified into susceptible and non-susceptible, per the guidelines of CLSI 2018 [44]. The susceptible and non-susceptible isolates were represented by 0 and 1, respectively, to determine the predictor variables (States, udder quarters, bacterial etiological agents, farms) of the non-susceptibility to antimicrobials by GLM-GEE analysis as described above. In all analyses, SPSS ver. 26 (SPSS Inc., Chicago, IL, USA) was used. The significance level was decided by assuming a 95% confidence interval at 5% alpha for the *p*-value cut-off. 

## 3. Results

### 3.1. Bovine Mastitis by the Three States, Udder Quarters and Causative Bacterial Species

A total of 193 bacteria isolates were obtained from 151 cows, of which 88 cows (58.3%) had subclinical mastitis, while 63 cows (41.7%) had clinical mastitis (Table 1 and Table 2). A total of 116 (60.1%) and 77 (39.9%) bacteria were isolated from subclinical and clinical cases of mastitis, respectively (Table 2). Of 174 total udder quarters with mastitis, 47 (27%) was in the left front (LF), 37 (21.3%) in the left rear (LR), 52 (29.9%) in the right front (RF), and 38 (21.9%) was in the right rear (RR) quarters (Table 2). Similarly, a total of 193 bacteria from LF (*n* = 53), LR (*n* = 37), RF (*n* = 62) and RR (*n* = 41) quarters were isolated and identified (Table 2). The majority of *Staph. aureus* (90.9%), *K. pneumoniae* (61.5%), and *Strep. uberis* (7.5%) were isolated from subclinical mastitis, whereas the majority of *E. coli* (79.4%), *K. oxytoca* (75.0%), and *Strep. dysgalactiae* (52.8%) were isolated from clinical mastitis (Table 1 and Table 2). The overall percentage of isolated bacteria involved in the occurrence of mastitis was not significantly different among three US states (*p* = 0.964) and udder quarters (*p* = 0.39) but significantly different (*p* = 0.000) among bacterial causative agents reported in this study (Table 1 and Table 2). Accordingly, the major causative bacterial species isolated from cases of bovine mastitis in this study area in descending order included *Staph. aureus* (34.2%), *Strep. uberis* (20.7%), *Strep. dysgalactiae* (18.7%), *E. coli* (17.6%), *K. pneumonia* (6.7%), and *K. oxytoca* (2.1%) (Table 2).

**Figure 1:**
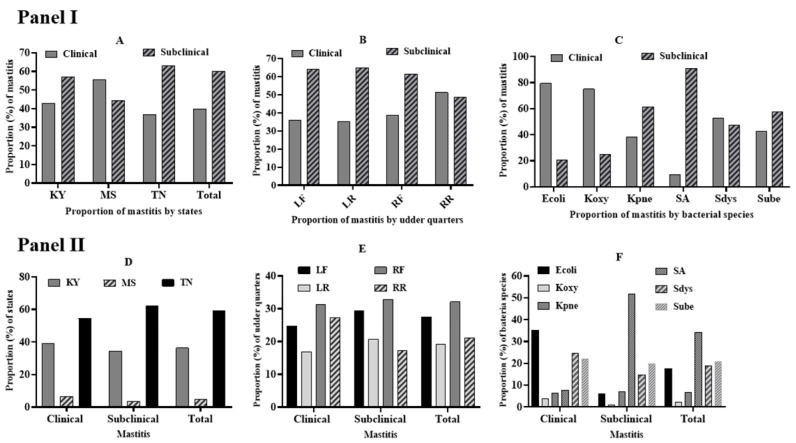
The distribution in the percentage of clinical and subclinical bovine mastitis within (Panel I) and among (panel II) three states, udder quarters with etiological bacterial species. Panel I showed the distribution of clinical and subclinical mastitis within three states, udder quarters, and etiological bacterial species. Panel I, (**A**) distribution of clinical and subclinical mastitis within each state, Panel I, (**B**) distribution of clinical and subclinical mastitis within each quarter, Panel I, (**C**) distribution of clinical and subclinical mastitis caused by each bacterial species, Panel II showed the distribution of clinical and subclinical bovine mastitis among three states, four udder quarters, and six etiological bacterial species. Panel II, (**D**) distribution of clinical and subclinical mastitis among three US states, Panel II, (**E**) distribution of clinical and subclinical mastitis among four udder quarters, Panel II, (**F**) distribution of clinical and subclinical mastitis among six bacterial species, Ky: Kentucky, MS: Mississippi, TN: Tennessee, LF: Left front, LR: Left rear, RF: Right front, RR: Right rear, Ecoli: *E. coli*, Koxy: *K. oxytoca*, Kpne: *K. pneumoniae*, SA: *Staph. aureus*, Sdys: *Strep. dysgalactiae*, Sube: *Strep. uberis*.

The proportion of clinical mastitis was not significantly different from subclinical mastitis in each state (Figure 1A). Further stratification of mastitis by udder quarters indicated a higher proportion of subclinical mastitis than clinical mastitis in all three quarters except right rear quarters, where the proportion of clinical mastitis was barely higher than subclinical mastitis (Figure 1B). The proportion of clinical mastitis due to *E. coli* or *K. oxytoca* was significantly higher than its respective subclinical mastitis, whereas the proportion of subclinical mastitis due to *Staph. aureus* was significantly higher than its clinical mastitis (Figure 1C). This was expected since Gram-negative bacterial mastitis pathogens are mainly known to cause more clinical mastitis than subclinical mastitis. There was no significant difference among USA states in the counts of clinical or subclinical mastitis (χ^2^ = 1.68; *p* = 0.431 (Figure 1D). Although both left and right front udder quarters were more affected by clinical and subclinical mastitis than left and right rear quarters (Figure 1E), the difference was not statistically significant (χ^2^ = 2.88; *p* = 0.41). In line with the observation shown by Figure 1C, a significantly higher proportion of *E. coli* (*p* < 0.001) and *Staph. aureus* (*p* < 0.05) were involved in clinical and subclinical mastitis, respectively (Figure 1F). Finally, we conducted a multivariable logistic regression model with all variables included in the model to factor out their confounding effect. The model indicated that *E. coli* and *Staph. aureus* were significant variables for predicting the occurrence of bovine mastitis among the explanatory variables (Table 3).

### 3.2. Antimicrobial Resistance Patterns of Bacterial Species Isolated from Cases of Mastitis

**Figure 2:**
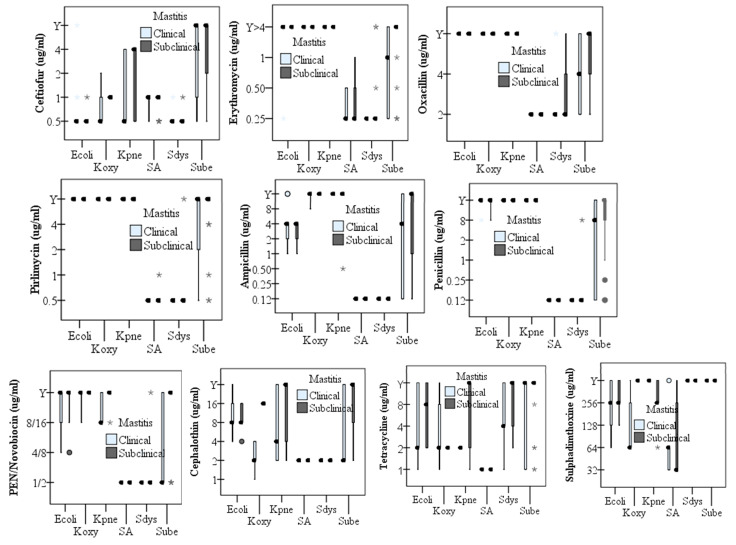
Box plot of minimum inhibitory concentration (MIC) values (µg/mL) of six bacterial species isolated from clinical and subclinical bovine mastitis against commonly used antimicrobials in dairy farms. In the box plot, the median MIC values are shown by dark dots and the standard error is shown by a vertical bar. The star symbol and white dots showed isolates with outlier MIC values. Ecoli: *E. coli*, Koxy: *K. oxytoca*, Kpne: *K. pneumoniae*, SA: *Staph. aureus*, Sdys: *Strep. dysgalactiae*, Sube: *Strep. uberis*. The “Y” value on the Y-axis was for isolate with MIC value above the maximum antimicrobial concentration coated on the Sensititre plate. The dark dots indicated the median MIC value, which corresponds to half (50%) of the isolates for respective bacterial species isolated from clinical or subclinical mastitis. For penicillin, penicillin-novobiocin, erythromycin, oxacillin, and pirlimycin all Gram-negative isolates had MIC value above the concentration of the antimicrobials coated on the Sensititre plate (Y > 4 µg/mL) because of their intrinsic resistance to these antimicrobials.

The six bacterial species had significantly different (*p* < 0.05) AMR against multiple antimicrobials used for the treatment of mastitis caused by both Gram-positives (*n* = 142) and Gram-negatives (*n* = 51), including cephalosporin, tetracycline, and sulfadimethoxine (Figure 2). The proportion of resistant isolates was relatively high in Klebsiella spp. and *E. coli* followed by Streptococcus spp. but low among *Staph. aureus* isolates. All *Staph. aureus* and *Strep. dysgalactiae* isolates were susceptible to ampicillin, cephalothin, and ceftiofur. All *Staph. aureus* isolates were also susceptible to penicillin, oxacillin, pirlimycin but resistant to tetracycline (Table 4).

### 3.3. Distribution of AMR Bacterial Isolates within Six Causative Bacterial Agents from Cases of Bovine Mastitis

AMR was relatively more widespread in *Klebsiella* spp. and *E. coli* isolates, followed by *Streptococcus* spp. than among *Staph. aureus* isolates (Table 4). The proportion of AMR bacterial isolates appeared variable among three USA states, clinical, subclinical mastitis, and bacterial species (Figure 2, Table 4), serving as explanatory variables for the variation. Therefore, we included these variables (states, clinical mastitis, subclinical mastitis and bacterial species) in multivariable logistic regression analysis to determine the significant risk factor (predictor) for the observed AMR against each of ten antimicrobials tested (Table 5). After controlling the confounding effects of states, type of mastitis, and bacterial species using Tennessee, *Strep. uberis*, and subclinical mastitis as a reference, bacterial isolates were compared for significant (*p* < 0.05) difference. Gram-negative bacterial isolates had intrinsic resistance against common penicillin, oxacillin, erythromycin, and pirlimycin, so these drugs are not effective against *Klebsiella* spp. and *E. coli.* The higher resistance observed in clinical, rather than subclinical mastitis, can be due to higher Gram-negative bacteria involvement in clinical mastitis in this study (Figure 1). *Escherichia coli* was significantly (*p* < 0.015) more susceptible to ampicillin, tetracycline, and ceftiofur than *Strep. uberis*. *Streptococcus dysgalactiae* was also significantly (*p* < 0.05) more susceptible to penicillin, erythromycin, oxacillin, pirlimycin, and penicillin/novobiocin but more resistant to tetracycline than *Strep. uberis*. *Staphylococcus aureus* and *K. pneumniae* were significantly (*p* < 0.011) more susceptible to erythromycin and tetracycline, respectively, than *Strep. uberis* (Table 5). 

## 4. Discussion

Six bacterial etiologic agents of bovine mastitis were isolated and identified, which include; *Staph. aureus*, *Strep. uberis*, *Strep. dysgalactiae*, *E. coli*, *K. pneumoniae*, and *K. oxytoca*. At the individual cow level, more cows had subclinical mastitis (58.3%) than clinical mastitis (41.7%). Studies elsewhere showed that subclinical mastitis is responsible for over 90% of the total loss of milk production [1]. The higher subclinical and clinical mastitis finding in this study should not surprise the readers because the sampling criteria used in this study were cows with SCC > 200,000 cells/mL of milk and cows that develop clinical mastitis during study time.

In this study, the majority of *E. coli* isolates were associated with clinical mastitis, whereas most *Staph. aureus* isolates were from subclinical mastitis, although other studies reported inconsistent findings concerning pathogens that were uniquely linked to subclinical and clinical mastitis. Accordingly, *Staph. aureus*, non-aureus staphylococci, *Strep. dysgalactiae*, *Strep. uberis*, *E. coli*, and *Streptococcus* spp. were linked to subclinical bovine mastitis [45,46], but *Staph. aureus*, *E. coli*, *Strep. uberis*, and non-aureus staphylococci were linked to clinical mastitis in Canada [47]. However, in Estonia, *Strep. uberis* and *E. coli* were linked to clinical mastitis, whereas *Staph. aureus* and non-aureus *Staphylococcus* were linked to subclinical mastitis [48]. Similar to our current findings, *Staph. aureus* was reported to be linked to subclinical mastitis in Germany [49] and Estonia [48]. This suggests considerable global differences among countries, farms, and even among studies at different times within a farm concerning the pathogens responsible for clinical and subclinical mastitis.

In this study, the distribution of bovine mastitis pathogens was not significantly different among three USA states and udder quarters but significant variations exist among bacterial species. However, other studies reported regional variations [50,51].

A relatively higher proportion of clinical and subclinical mastitis was observed in front quarters than rear quarters in this study. Anatomically the front quarter is significantly longer and wider at the teat apex than the rear quarter, which may predispose front quarters to higher infection rates than the rear quarters [52,53]. Both dirt and severe hyperkeratosis of the teat-end predispose the udder to clinical *E. coli* mastitis, whereas both severe hyperkeratosis and teat-ends with no callosity ring increase the risk of clinical *Strep. uberis* mastitis [54]. However, a previous study reported higher infection in rear quarters than in front quarters [55].

The proportion of resistant isolates was relatively high in *Klebsiella* spp. and *E. coli* followed by *Streptococcus* spp. but low among *Staph. aureus* isolates. Similar findings were reported from dairy farms in Canada [56]. Contrary to our findings, in Sweden, the majority of *Staph. aureus*, non-aureus staphylococci, *Strep. uberis*, *Strep. dysgalactiae*, *Strep. agalactiae*, *E. coli*, and *Klebsiella* spp. isolates were susceptible to most antimicrobials, except penicillin [57]. The widespread AMR in *Klebsiella* spp. and *E. coli* isolates, followed by *Streptococcus* spp. and low AMR among the *Staph. aureus* isolates. Gram-negative bacterial isolates have intrinsic resistance to common penicillin, oxacillin, erythromycin, pirlimycin, and penicillin-novobiocin so *E. coli* and *Klebsiella* spp. were not sensitive to these antimicrobials. The outer membrane of Gram-negative bacteria has two barriers for antimicrobials to reach their target in intracellular processes, which include the lipid-mediated barriers that block hydrophilic antimicrobials and the general diffusion porins that block hydrophobic antimicrobials [58]. Among the Gram-negative bacteria, *Klebsiella pneumoniae* is also a well-recognized antimicrobial-resistant pathogen in human medicine [59]. *Klebsiella pneumoniae* [60] is considered as a super-resistant pathogen and Trojan-horse that acquires, carries and spreads different AMR genes from the environment to pathogens of livestock and humans. Thus, the observed resistant *K. pneumoniae* and *E. coli* in bovine mastitis in this study could be a threat to the dairy industry and public health since the sharing of AMR genes and transfer among Gram-negative and Gram-positive bacteria are already reported [60,61].

The six bacterial species had a significantly different (*p* < 0.05) proportion of resistance against multiple antimicrobials used for the treatment of mastitis caused by both Gram-positives and Gram-negatives, including cephalosporin, tetracycline, and sulfadimethoxine. Based on this study, Gram-negatives are more resistant than Gram-positives but whether this difference is due to intrinsic resistance of Gram-negatives to some of these antimicrobials or due to the development of resistance under the presence of antimicrobials prescriptions need further investigation. The fitness to thrive under antimicrobials may be acquired and promoted due to the frequent imprudent use of antimicrobials in dairy farms in the study area since an unregulated imprudent antimicrobial use pattern was reported among dairy farmers in Tennessee [62]. However, we do not have antimicrobials use patterns of farms included in this study to support this finding. Generally, over 85% of US dairy farms treat cows with antimicrobials for mastitis, and over 90% of them use intramammary antimicrobials at dry off [63], and 80% of them treat all cows on the farm [64]. Such imprudent use might induce pressure on bacteria in the gastrointestinal tract and other parts of an animal body, but there are minimal data concerning the monthly and yearly dynamics of antimicrobial type and dose that farmers commonly use to substantiate that farmers imprudently use antimicrobials to find reasons for expanding AMR pathogens in dairy production. A study in New Zealand indicated that the type, dose, and intensity of antimicrobials use practice fluctuates from one antimicrobial to another in different herds, regions, years, and months [65]. A recent report in Tennessee indicated that the preferential consumption of the medically important antimicrobial classes based on the frequency of prescriptions was as follows: cephalosporins > penicillins > tetracyclines > fluoroquinolones > sulfa > aminoglycosides > macrolides > lincosamides, but these preferences varied by years of experience in clinical practice, year of graduation and concern for AMR by the practicing veterinary clinician [66,67]. However, there was no data for predictive modeling concerning to what extent the fluctuations in antimicrobial type, dose, intensity, and preference for a prescription (use) contribute to changes in the prevalence of AMR. Therefore, further research is required to determine factors that drive the development of antimicrobial resistance in dairy farms. Further investigation will also be needed to predict seasonal, farm, and state-level variations in the type of antimicrobials used in relation to the AMR patterns. 

The number of farms, which were willing to participate, was higher in Tennessee than Kentucky and Mississippi. Subsequently, the number of cows and bacteria isolates collected from KY and MS were low. Therefore, readers should interpret our findings cautiously based on this limitation. 

## 5. Conclusions

Six bacterial etiologic agents of bovine mastitis were isolated and identified. There was a higher proportion of subclinical mastitis than clinical mastitis. Antimicrobial-resistant Gram-positive and Gram-negative bacterial mastitis pathogens were abundant in milk collected from cows with SCC > 200,000 cells/mL and cows with clinical mastitis. Therefore, continuous monitoring of AMR and application of AMR mitigation measures are required to control their spread to humans, animals, and the environment.

## Figures and Tables

**Table 1:** Cows with mastitis (*n* = 151) in 34 dairy farms from Kentucky, Mississippi, and Tennessee.

	Ecoli	%	Koxy	%	Kpne	%	SA	%	Sdys	%	Sube	%	Total
KY (*n* = 70 isolates)	15	21.4			9	12.9	16	22.9	19	27.1	11	15.7	70
MS (*n* = 9 isolates)	3	33.3							1	11.1	5	55.6	9
TN (*n* = 114 isolates)	16	14.0	4	3.5	4	3.5	50	43.9	16	14.0	24	21.1	114
Cows with mastitis (*n* = 151)	32	21.2	3	2.0	12	7.9	46	30.5	32	21.2	31	20.5	151
Farms with mastitis (*n* = 34)	15	44.1	3	8.8	7	20.6	15	44.1	19	55.9	21	61.8	

KY: Kentucky, MS: Mississippi, TN: Tennessee, Ecoli: *E. coli*, Koxy: *Klebsiella oxytoca*, Kpne: *K. pneumoniae*, SA: *Staphylococcus aureus*, Sdys: *Streptococcus dysgalactiae*, Sube: *Streptococcus uberis*.

**Table 2:** Bacterial isolates (*n* = 193) from clinical and subclinical cases of bovine mastitis in three states.

	Ecoli		Koxy		Kpne		SA		Sdys		Sube		Total isolates	
Measurement	*n* = 34	%	*n* = 4	%	*n* = 13	%	*n* = 66	%	*n* = 36	%	*n* = 40	%	N = 193	%
LF (*n* = 47)	10	29.4%	3	75.0%	0	0.0%	16	24.2%	11	30.6%	13	32.5%	53	27.5%
LR (*n* = 37)	5	14.7%	0	0.0%	5	38.5%	14	21.2%	7	19.4%	6	15.0%	37	19.2%
RF (*n* = 52)	9	26.5%	0	0.0%	5	38.5%	23	34.8%	12	33.3%	13	32.5%	62	32.1%
RR (*n* = 38)	10	29.4%	1	25.0%	3	23.1%	13	19.7%	6	16.7%	8	20.0%	41	21.2%
Clinical mastitis (*n* = 63)	27	79.4%	3	75.0%	5	38.5%	6	9.1%	19	52.8%	17	42.5%	77	39.9%
Subclinical mastitis (*n* = 88)	7	20.6%	1	25.0%	8	61.5%	60	90.9%	17	47.2%	23	57.5%	116	60.1%
Share of total isolates (*n* = 193)	34	17.6%	4	2.1%	13	6.7%	66	34.2%	36	18.7%	40	20.7%		

LF: Left front, LR: Left rear, RF: Right front, RR: Right rear, Ecoli: *E. coli*, Koxy: *Klebsiella oxytoca*, Kpne: *K. pneumoniae*, SA: *Staphylococcus aureus*, Sdys: *Streptococcus dysgalactiae*, Sube: *Streptococcus uberis*.

**Table 3:** Multivariable logistic regression analysis for predicting the occurrence of bovine mastitis using some explanatory variables.

Parameter	B	Std. Error	95% Wald CI		Test			Exp (B)	95% Wald CI for Exp (B)	
			Lower	Upper	Wald χ^2^	df	Sig.		Lower	Upper
(Intercept)	−0.298	0.7365	−1.742	1.146	0.164	1	0.686	0.742	0.175	3.144
State										
KY	0.199	0.8039	−1.377	1.775	0.061	1	0.805	1.22	0.252	5.897
MS	0.242	1.2597	−2.227	2.712	0.037	1	0.847	1.274	0.108	15.052
TN	Ref							1		
Udder quarter										
LF	0.957	0.5729	−0.166	2.08	2.79	1	0.095	2.604	0.847	8.003
LR	0.529	0.4999	−0.451	1.508	1.118	1	0.29	1.696	0.637	4.519
RF	0.411	0.4642	−0.499	1.321	0.783	1	0.376	1.508	0.607	3.746
RR	Ref							1		
Bacterial species										
*Ecoli*	−1.676	0.6837	−3.016	−0.335	6.006	1	0.014	0.187	0.049	0.715
*Koxy*	−1.562	1.1707	−3.856	0.733	1.78	1	0.182	0.21	0.021	2.08
*Kpne*	0.274	0.8581	−1.408	1.956	0.102	1	0.749	1.315	0.245	7.07
*SA*	2.108	0.4882	1.152	3.065	18.653	1	0.000	8.234	3.163	21.435
*Sdys*	−0.46	0.5188	−1.477	0.556	0.788	1	0.375	0.631	0.228	1.744
*Sube*	Ref							1		

KY: Kentucky, MS: Mississippi, TN: Tennessee, RF: Right front, RR: Right rear, LR: Left rear, LF: Left front, Ecoli: *E. coli*, SA: *Staphylococcus aureus*, Sube: *Streptococcus uberis*, Sdys: *Streptococcus dysgalactiae*, Koxy: *K. oxytoca*, Kpne: *K. pneumoniae*, 95% Wald CI: the 95% Wald confidence interval; B: is logarism value, Exp (B): the anti-log of B which is the values for the logistic regression equation for predicting the dependent variable, from the independent variable. 95% Wald CI for Exp (B): the 95% confidence interval of B.

**Table 4:** Significance of the proportion (%) of antimicrobial resistant bacterial species from cases of bovine mastitis. Ecoli (*n* = 34), Koxy (*n* = 4), Kpne (*n* = 13), SA (*n* = 66), Sdys (*n* = 36), and Sube (*n* = 40).

Antimicrobial	*Bacterial* spp.	No. AMR Isolate	Percentage (%)	95% CI Lower	95% CI Upper	Sig.
AMP	Ecoli (*n* = 34)	2	5.9	0.7	19.7	0.000
	Koxy (*n* = 4)	3	75	19.4	99.4	0.625
	Kpne (*n* = 13)	12	92.3	64	99.8	0.003
	SA (*n* = 66)	0	0	0	5.4	0.000
	Sdys (*n* = 36)	0	0	0	9.7	0.000
	Sube (*n* = 40)	28	70	53.5	83.4	0.018
PEN	SA	0	0	0	5.4	0.000
	Sdys	2	5.6	7	18.7	0.000
	Sube	31	77.5	61.5	89.2	0.001
ERY	Ecoli	33	97.1	84.7	99.9	0.000
	Koxy	4	100	39.8	100	0.125
	Kpne	13	100	75.3	100	0.000
	SA	1	1.5	0	8.2	0.000
	Sdys	2	5.6	7	18.7	0.000
	Sube	32	80	64.4	90.9	0.000
OXA	SA	0	0	0	5.4	0.000
	Sdys	8	22.2	10.1	39.2	0.002
	Sube	27	67.5	50.9	81.4	0.04
PRL	SA	0	0	0	5.4	0.000
	Sdys	1	2.8	0.1	14.5	0.000
	Sube	32	80	64.4	90.9	0.000
	SA	0	0	0	5.4	0.000
	Sdys	2	5.6	0.7	18.7	0.000
	Sube	27	67.5	50.9	81.4	0.04
TET	Ecoli	14	41.2	24.6	59.3	0.391
	Koxy	1	25	0.6	80.6	0.625
	Kpne	5	38.5	13.9	68.4	0.581
	SA	66	100	0	5.4	0.000
	Sdys	34	94.4	81.3	99.3	0.000
	Sube	31	77.5	61.5	89.2	0.0001
CEPH	Ecoli	11	32.4	17.4	50.5	0.059
	Koxy	1	25	0.6	80.6	0.625
	Kpne	7	53.8	25.1	80.8	1
	SA	0	0	0	5.4	0.000
	Sdys	0	0	0	9.7	0.000
	Sube	25	62.5	45.8	77.3	0.155
CFT	Ecoli	1	2.9	0.1	15.3	0.000
	Koxy	4	100	0	60.2	0.125
	Kpne	7	53.8	25.1	80.8	1
	SA	0	0	0	5.4	0.000
	Sdys	0	0	0	9.7	0.000
	Sube	25	62.5	45.8	77.3	0.155
SULPH	Ecoli	10	29.4	15.1	47.5	0.026
	Koxy	2	50	6.8	93.2	1
	Kpne	7	53.8	25.1	80.8	1
	SA	10	15.2	7.5	26.1	0.000
	Sdys	36	100	90.3	100	0.000
	Sube	40	100	91.2	100	0.000

Ecoli: *E. coli*, SA: *Staphylococcus aureus*, Sube: *Streptococcus uberis*, Sdys: *Streptococcus dysgalactiae*, Koxy: *K. oxytoca*, Kpne: *K. pneumoniae*, AMP: ampicillin, PEN: penicillin, ERY: erythromycin, OXA: oxacillin, PRL: pirlimycin, P/N: penicillin-novobiocin, TET: tetracycline, CEPH: cephalothin, CFT: ceftiofur, SULPH: sulfadimethoxine, No AMR isolates: number of antimicrobial resistant isolates, CI: confidence interval, Sig: significance.

**Table 5:** Multivariable logistic regression analysis of the strength of association between potential risk factors and occurrence of AMR to ten antimicrobials among six bacteria species isolated from bovine mastitis.

Antimicrobials	Predictor Variable/Reference	B	S.E.	Wald	df	Sig.	Exp (B)	95% CI Lower EXP (B)	95% CI Upper EXP (B)
Ampicillin	Ecoli/Sube	−3.613	0.87	17.266	1	0.000	0.027	0.005	0.148
Penicillin	Sdys/Sube	−4.314	0.899	23.001	1	0.000	0.013	0.002	0.078
Erythromycin	Clinical/Subclinical	−1.544	0.779	3.931	1	0.047	0.213	0.046	0.983
Erythromycin	EC/Sube	2.808	1.154	5.921	1	0.015	16.57	1.726	159.039
Erythromycin	SA/Sube	−6.188	1.196	26.786	1	0.000	0.002	0	0.021
Erythromycin	Sdys/Sube	−4.343	0.904	23.067	1	0.000	0.013	0.002	0.076
Oxacillin	Sdys/Sube	−2.04	0.581	12.312	1	0.000	0.13	0.042	0.406
Pirlimycin	Sdys/Sube	−5.644	1.346	17.574	1	0.000	0.004	0	0.05
PEN/NOVO	Clinical/Subclinical	−1.418	0.695	4.162	1	0.041	0.242	0.062	0.946
PEN/NOVO	Sdys/Sube	−3.92	0.917	18.272	1	0.000	0.02	0.003	0.12
Tetracycline	EC/Sube	−1.376	0.55	6.253	1	0.012	0.253	0.086	0.743
Tetracycline	Kpne/Sube	−1.873	0.738	6.437	1	0.011	0.154	0.036	0.653
Tetracycline	Sdys/Sube	1.698	0.84	4.092	1	0.043	5.464	1.054	28.321
Ceftiofur	EC/Sube	−3.989	1.094	13.305	1	0.000	0.019	0.002	0.158

PEN/NOVO: Penicillin/novobiocin, Ecoli: *E. coli*, SA: *Staphylococcus aureus*, Sube: *Streptococcus uberis*, Sdys: *Streptococcus dysgalactiae*, Koxy: *Klebsiella oxytoca*, Kpne: *Klebsiella pneumoniae*, S.E: Standard error, df: degree of freedom; B: is logarism value, Exp (B): the anti-log of B, which is the logistic regression equation’s values for predicting the dependent variable, from the independent variable. 95% Wald CI for Exp (B): the 95% confidence interval of B.

## Data Availability

Data sharing not applicable- No new data were created or analyzed in this study. Data sharing is not applicable to this article.
